# Antimicrobial resistance genes in weaned pigs: quantitative abundance and group dynamics assessed by qPCR

**DOI:** 10.3389/fvets.2025.1709227

**Published:** 2026-01-05

**Authors:** Megarsa Jaleta, Vera Junker, Christina Hölzel, Jürgen Zentek, Thomas Amon, Ulrich Nübel, Tina Kabelitz

**Affiliations:** 1 Leibniz Institute for Agricultural Engineering and Bioeconomy (ATB), Potsdam, Germany; 2 Leibniz-Institute DSMZ – German Collection of Microorganisms and Cell Cultures, Braunschweig, Germany; 3 Faculty of Agricultural and Nutritional Sciences, Kiel University, Kiel, Germany; 4 Institute of Animal Nutrition, Freie Universität Berlin, Berlin, Germany; 5 Institute for Animal Hygiene and Environmental Health (ITU), Freie Universität Berlin, Berlin, Germany; 6 German Center for Infection Research (DZIF), Partner Site Braunschweig-Hannover, Braunschweig, Germany; 7 Technical University Braunschweig, Institute of Microbiology, Braunschweig, Germany

**Keywords:** ARG, antimicrobial resistance, qPCR, SmartChip, weaner pigs

## Abstract

Antibiotic resistance genes (ARGs) linked with the selection of resistant bacteria in intensive commercial livestock production require regular surveillance. This study quantified ARG abundances in weaner pigs, with emphasis on inter-individual variation and temporal trends of ARG dynamics over 8 weeks in a flat-deck housing system. Fecal samples from 103 individual pigs and 53 pooled pen-floor collections were analyzed. Broad-spectrum ARG profiling of pooled DNA from both sample types was performed using high-throughput qPCR (SmartChip), while standard qPCR quantified eight ARGs (*aadA1*, *bla*_TEM_, *dfrA12*, *erm*(B), *lnu*(F), *qnrS*, *sul2*, and *tet*(A)) and the *16S rRNA* gene in all samples. Among the quantified ARGs, *erm*(B) and *aadA1* were the most abundant, while *qnrS* was least frequently detected. Substantial inter-individual variation was observed for most ARGs, despite pigs living together under the same management conditions. Pooled pen-floor feces exhibited significantly higher ARG loads than individual fecal samples, suggesting that resistome profiles at the pen-floor level cannot be accurately inferred from fresh feces of individual animals. Temporal analysis revealed that *16S rRNA* gene copies increased during the later period, while *tet*(A) and *sul2* decreased, suggesting age-related effects. These findings reveal pronounced intra-cohort variability in ARG abundance among weaner pigs and underscore the impact of weaning-associated factors on resistome composition. Future investigations should examine the contribution of gut microbiome dynamics and evaluate dietary interventions aimed at stabilizing ARG profiles and mitigating the resistance development of microbial resistance.

## 1 Introduction

Pig production represents the second largest source of meat worldwide, following poultry (1). The use of antimicrobial drugs for the treatment of diseases is widespread in intensive commercial systems and underpins large-scale meat production (2). Regular monitoring of antimicrobial resistance (AMR) in livestock is critical, as certain bacteria serve as reservoirs and can transmit resistance to humans through multiple pathways (3–5).

In pig production systems, numerous studies consistently demonstrate that weaners represent the most intensively treated age group across countries, based on treatment frequency. Although the dominant antimicrobial classes vary by region, data from Germany and several European countries before 2019 indicate that penicillins, polymyxins, and tetracyclines were the most frequently used drug classes (6–11). However, the use of polymyxins (colistin) has been declining consistently over time, as they are recommended to be reserved as a last-resort agents for critical human infections (6). The dependency on these drugs may be associated with several factors that render weaner pigs particularly vulnerable to diseases, including weaning stress, immature immune system development, environmental factors, early dietary transitions, and intestinal microbiome dysbiosis (12–14). Since 28 January 2022, routine antimicrobial use has been prohibited, and they may no longer be employed to compensate for poor hygiene, inadequate husbandry, or lack of care (15).

Surveillance of AMR traditionally focused on detecting AMR in specific indicator bacteria, such as *Escherichia coli*, using antimicrobial susceptibility testing and whole-genome sequencing, with results typically reported as prevalence or incidence. However, culture-based isolation of fecal bacteria is both labor-intensive and inherently selective, leaving several bacterial species uncultured (16, 17). Dependence on culture-based methods may result in an incomplete assessment of the resistome, as cultivation alone does not fully capture the abundance and distribution of ARGs within a sample.

This gap can be filled through the molecular methods, particularly metagenomic analysis and/or quantitative polymerase chain reaction (qPCR), to profile ARGs in complex environmental samples such as feces. Both approaches effectively detect resistance genes; however, qPCR offers higher sensitivity for specific ARGs and enables precise quantification, allowing robust comparisons between individual and pooled samples while capturing inter-individual variation (18–21). In contrast, metagenomics sequencing (MGS) offers greater specificity, reduced off-target binding, and the capacity to identify multiple ARG subtypes that standard qPCR cannot distinguish. Nonetheless, high-throughput qPCR platforms, such as the SmartChip system, enable parallel detection of numerous ARG subtypes using arrays of primer pair sets, thereby strengthening ARGs surveillance in complex environmental samples (21–23).

In studies of ARGs in weaner pigs, many investigations have focused on experimentally treated animals, evaluating the impact of antimicrobial agents on ARG abundance (24–28). Prior research has also emphasized longitudinal studies spanning the production cycle from farrowing to slaughter (14, 29); however, these studies have not examined ARG dynamics in weaner pigs in detail or at high temporal resolution. Moreover, little is known about how therapeutic antibiotics administered to individual sick animals influence ARGs at both the individual and herd level. Despite uniform housing and management conditions, individual weaner pigs exhibit substantial variation in AMR profiles (30). The present study therefore aimed to investigate the temporal dynamics and inter-individual variability of fecal ARGs in weaner pigs, assessing how ARG abundance and diversity change over time in both individual and herd-level samples.

## 2 Materials and methods

### 2.1 Study design

We tracked trends and patterns of ARGs in weaned pig feces on a weekly basis from the time they arrived in the weaner barn until they moved to the finisher barn for a period of 8 weeks. The study was carried out in a pig-fattening farm at the Teaching and Research Institute for Animal Breeding and Animal Husbandry (LVAT) e. V. Ruhlsdorf, State of Brandenburg, Germany.

**Figure 1:**
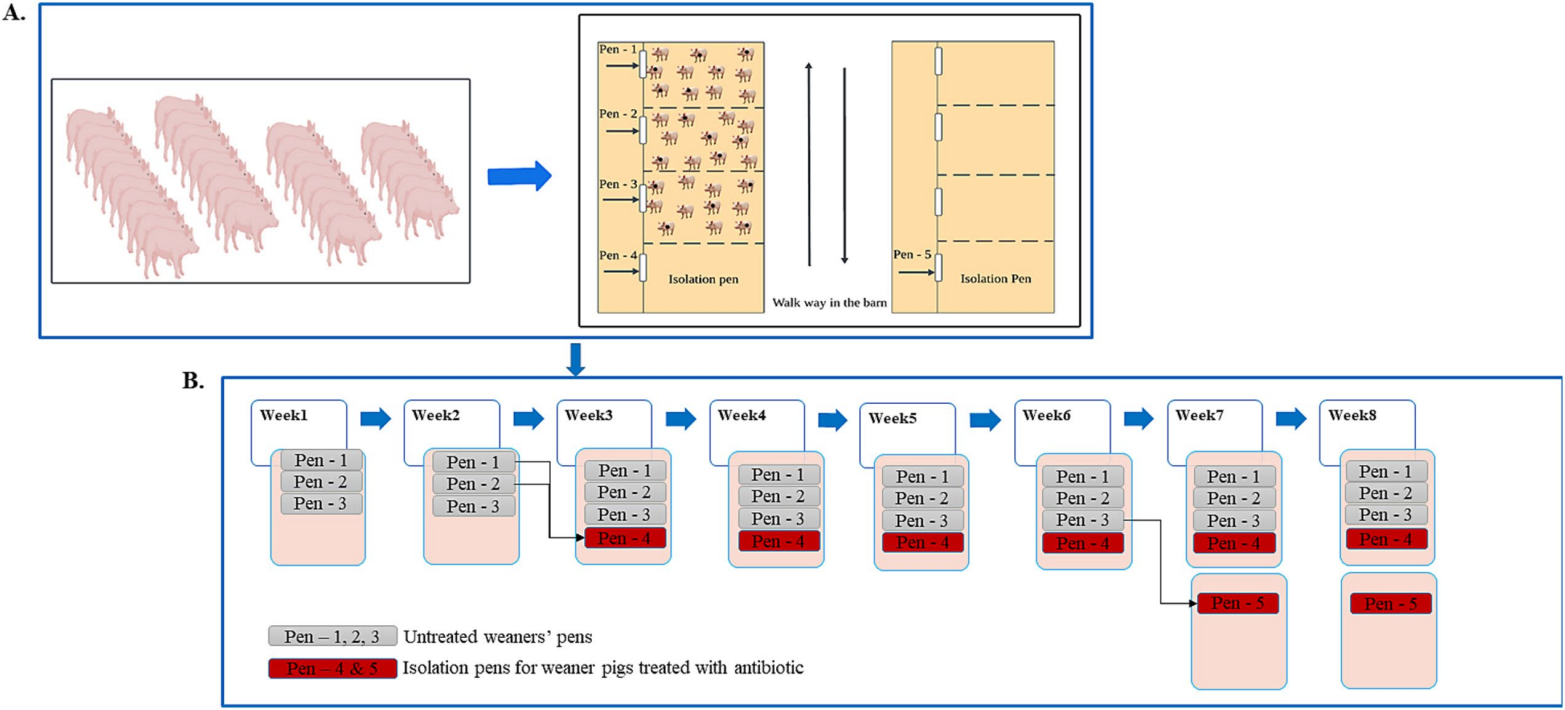
Schematic representation of the piglet’s placement in the flat deck. **(A)** A total of 33 weaned piglets of the same batch purchased from the same breeder were initially placed in three pens of clean and disinfected flat deck. Fifteen pigs were marked, and samples were collected from them individually for 8 weeks. **(B)** Schematic representation of the timing and location of movement of the treated pigs during the study period.

For our study, a batch of weaners aged 4 weeks (33 piglets in total) was purchased from one breeder and housed in a pre-cleaned and disinfected weaner flat deck with four identical pens. Approximately two-thirds of each pen had a metal slatted floor wrapped with perforated plastic material. The remaining third had a closed and heated floor serving as a lying area, covered with a roof and a rubber curtain to ensure a higher temperature in the lying area. Initially, the piglets were divided into three equal groups of 11, each group housed in one pen (Figure 1). In each of the three groups, five piglets were assigned (marked) and used as assigned animals for the collection of individual fecal samples. For all 15 selected animals, all medical information since birth was recorded. Information about farm management (feeding, cleaning, fly control, and medication strategies) was described in (31). Additional farm management information, including feeding and vaccination, are provided in the Supplementary material and Supplementary Table S6.

In this study, antibiotics were administered solely for therapeutic purposes to diseased pigs, as suggested by the farm veterinarian. During the farrowing period, a sow with 11 piglets out of 33 piglets recruited in our study received 8 mL of lincomycin antibiotic per day for 3 days at the breeder farm. After weaned pigs arrived at the fattening farm, 15 were selected as assigned pigs. Five of 15 selected pigs were treated with amoxicillin (Zoetis Deutschland GmbH, Berlin, Germany) at a dosage of 15 mg/kg for 3 days. The treatment was administered intramuscularly in the neck region, directly behind the ear, due to tail bite injuries. Two piglets were treated in the third study week, and the other three in the sixth week. Among the pigs not included in the assigned group, three were treated with amoxicillin and isolated in a separate pen on another empty flat-deck. No further monitoring was conducted for these pigs.

### 2.2 Sample and data collection

Freshly excreted feces from each assigned (marked) weaner pig and two pooled fecal samples per pen (to determine ARGs at the herd level) were collected weekly from April 4 to May 23, 2024. For the pooled feces from the pen floor, approximately 10–15 drops of fresh feces were collected from multiple locations within each pen using sterile spatula. The samples were transported with cooling to ATB and stored at −20 °C. After finishing the 8-week sampling period, a total of 156 fecal samples (103 from individual pigs and 53 from the herds) were transported on dry ice to the DSMZ laboratories and stored at −80 °C until DNA isolation.

### 2.3 Sample processing

#### 2.3.1 DNA extraction

For DNA extraction, approximately 0.2 grams of each fecal sample was measured, and extraction was performed using IHMS Protocol Q (International Human Microbiome Standards) as described in the Supplementary protocol (32). The extracted DNA concentrations ranged from 169.8 to 849.6 ng/μL, with acceptable purity levels (A260/A280: 1.82–1.88 for 95% of samples and 1.69–1.82 for the remainder), suitable for qPCR and measured by NanoDrop. Samples were stored at −20 °C until use.

#### 2.3.2 High-throughput ARG screening and qPCR validation

To obtain a preliminary overview of the ARGs present in the fecal samples, two pooled DNA extractions generated from the fecal samples on two sampling days and submitted in duplicate to Resistomap (Helsinki, Finland) for screening by high-throughput qPCR (33). This screening performed standard primer sets ARG2.1, which contained 384 primer pairs for diverse ARGs, integrons, mobile genetic elements (MGEs), bacterial taxa, and the *16S rRNA* gene. A detailed list of target genes for ARG2.1 in this SmartChip screening was provided in Supplementary Table S1.

To validate and quantify specific ARGs identified by a high-throughput SmartChip screening, a subset of ARGs was selected for targeted qPCR analysis in pig fecal samples. An initial panel of 15 ARGs was chosen based on SmartChip detection frequency and abundance, the farm’s antimicrobial usage profile, and the public health relevance of associated resistance mechanisms. Several primer sets, however, showed inconsistent amplification between the SmartChip and qPCR assays (Supplementary Table S2). Accordingly, eight ARGs (*aadA1*, *bla*_TEM_, *dfrA12*, *erm*(B), *lnu*(F), *qnrS*, *sul2*, and *tet*(A)) were retained for qPCR analysis. These genes encompass major antimicrobial classes used in swine production and include resistance determinants of clinical concern.

#### 2.3.3 qPCR quantification of selected resistance genes

Eight ARGs (*aadA1*, *bla*_TEM_, *dfrA12*, *erm*(B), *lnu*(F), *qnrS*, *sul2*, and *tet*(A)) and the *16S rRNA* gene (used for normalization as a proxy for total bacterial abundance) were quantified using a LightCycler 480 Instrument II (Roche Diagnostics Deutschland GmbH). Primer pairs were primarily selected from the literature based on similar previous studies, with additional primers derived from the standard ARG2.1 SmartChip panel (Resistomap, Finland), as listed in Supplementary Table S3. PCR products amplified from previously isolated positive strains were purified and used as standards and positive controls to enable absolute quantification. Each 20 μL reaction contained 10 μL SYBR Green Master I (LightCycler 480 SYBR Green I Master, Roche), 1 μL of each primer (10 μM), 6 μL PCR-grade nuclease-free water, and 2 μL DNA template. Reactions without DNA served as negative controls. The qPCR protocol comprised an initial denaturation at 95 °C for 180 s), and a final melting curve acquisition (95 °C for 5 s, 65 °C for 60 s, 97 °C, and cooling to 40 °C for 10 s). Absolute ARG copy numbers were calculated using standard curves derived from purified gene fragments of known concentration. Cycle threshold (Ct) values were interpolated against these curves, and resulting copy numbers were normalized to fecal mass to obtain ARG copies per gram of feces (Supplementary Table S4). Corresponding amplification parameters were also provided in Supplementary Table S3.

### 2.4 Statistical analysis

All analyses were performed in R v4.4.1 using tidyverse, ggpubr, performance, and lme4 packages. ARG abundances were normalized to *16S rRNA* gene copies (ARG/16S × 10^6^) and log₁₀-transformed for statistical comparison. Pigs were categorized as treated or untreated, and treated pigs were further evaluated pre- and post-treatment. Temporal effects were assessed by comparing early (weeks 1–4) vs. late (weeks 5–8) periods. Linear mixed-effects models (lme4) with pig ID as a random effect were used to quantify inter-individual variance, and intraclass correlation coefficients (ICC) were calculated (performance package) to determine the proportion of variance attributable to differences between pigs. Group comparisons (individual vs. pooled samples, treatment effects, feed-type effects) were performed using Wilcoxon signed-rank or rank-sum tests, and sow-level effects by Kruskal–Wallis tests. Statistical significance was set at *p* < 0.05. Missing samples due to observation constraints or animal relocation were assumed to be at random and were not imputed; analyses were conducted using all available data.

## 3 Results

### 3.1 Screening results of pooled sample DNA using Resistomap SmartChip

**Figure 2:**
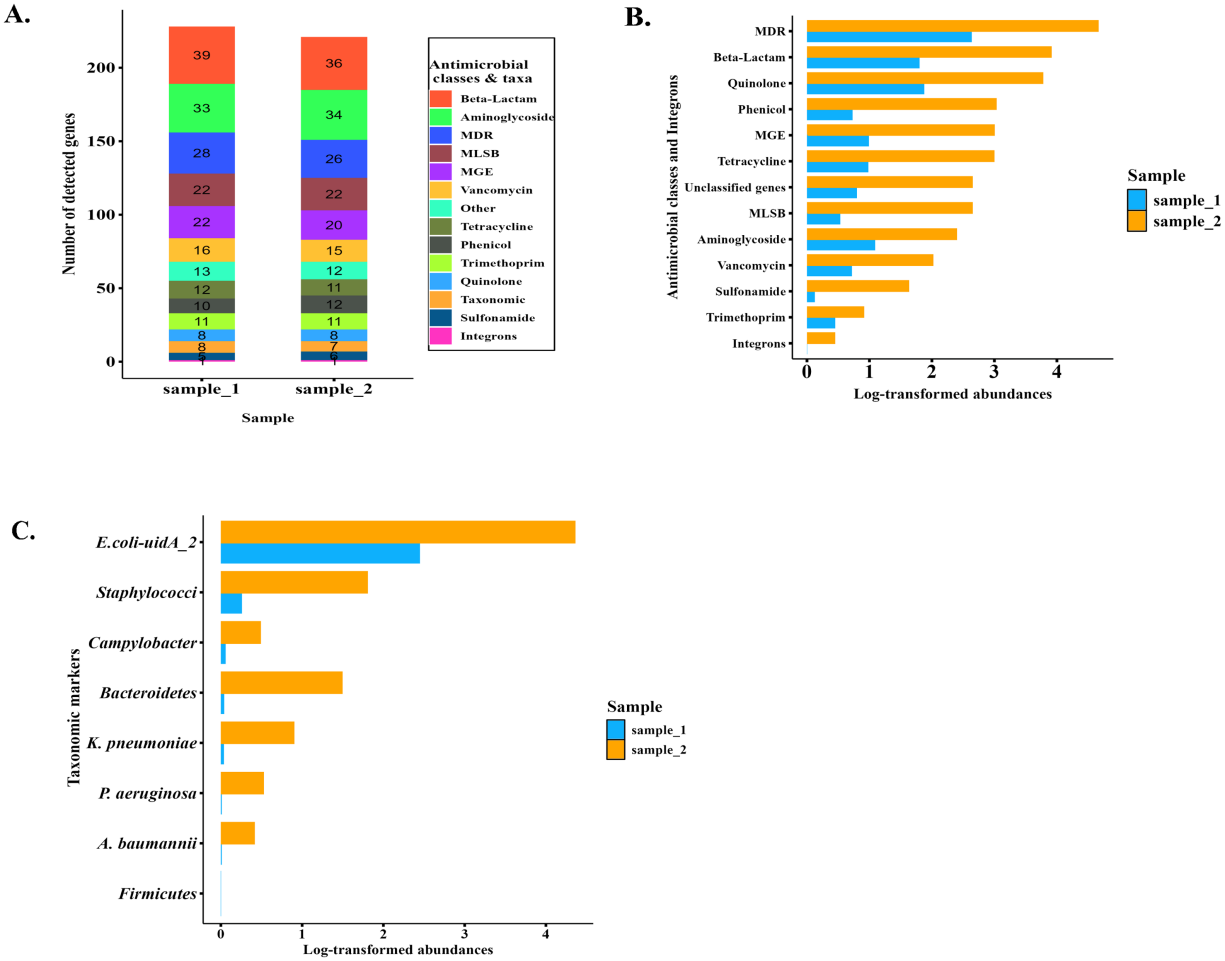
Antimicrobial resistance genes (ARGs), mobile genetic elements (MGEs), and bacterial taxonomic markers were quantified in two pooled weaner fecal DNA samples using a high-throughput SmartChip qPCR system. **(A)** Stacked bar plot showing the number of detected genes across antimicrobial resistance (AMR) classes and taxa per sample (numbers inside bars indicate total genes per category). **(B–C)** Relative abundances grouped based on AMR classes, MGEs, integrons **(B)**, and taxonomic markers **(C)**. Duplicate reactions were consistent, while profiles differed between samples, with Sample_2 depicting generally higher ARG burden and broader resistance diversity. MGE, Mobile genetic elements; MDR, Multidrug resistance.

SmartChip detected about 201 ARGs; *β*-lactams were the most prevalent (*n* = 39), followed by those against aminoglycosides (*n* = 34). In addition, taxonomic markers (*n* = 8), mobile genetic elements (MGEs; *n* = 22), and one integron gene were detected. The results of the screening (ARG relative abundance and gene copies) were downloaded and sequentially visualized as shown in Figure 2. Duplicated samples showed similar resistance gene profiles; the most dominant resistance genes detected by SmartChip are shown in Figure 2A. Individual gene abundances for most of the predominant quantified ARGs are presented in the heat maps included in the Supplementary materials (Supplementary Figure S1). MDR genes were the most abundant resistance genes, followed by *β*-lactam and quinolone resistance genes with relative abundance greater than 10^4^ (Figure 2B). Among nine specific bacterial taxonomic markers, screening indicates *E. coli* (*E. coli-uidA_2*) was highly abundant (Figure 2C).

### 3.2 Comparison of antimicrobial resistance detection by SmartChip and qPCR

SmartChip enabled broad screening of numerous ARGs, whereas qPCR provided precise quantification of selected genes in individual samples. Several ARGs, including *bla*_TEM_, *aadA1*, *qnrS*, *tet*(A), and *sul2*, were consistently detected by both SmartChip and targeted qPCR. However, SmartChip detected ARGs such as *mcr-1*, *bla*_CTX-M_, and *dfrA12* that were not identified by qPCR, whereas ARGs such as *tet*(B) and *bla*_TEM_ were weakly or not detected by SmartChip, highlighting method-specific discrepancies. These results highlight the importance of further research to clarify and reconcile differences between SmartChip screening and standard qPCR, thereby supporting accurate and comprehensive ARG surveillance.

### 3.3 Interindividual ARG abundance variation among a cohort of weaner pigs

**Figure 3:**
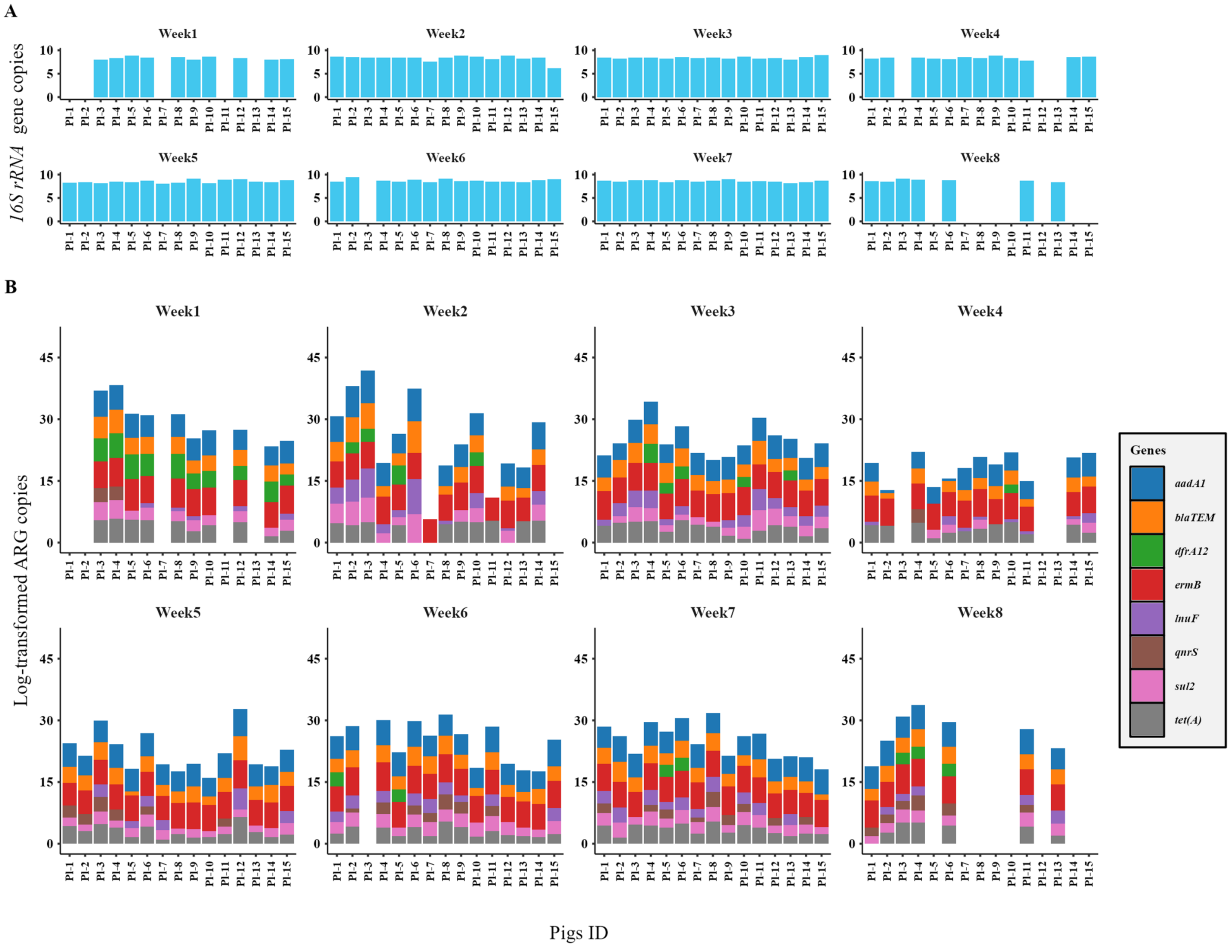
Temporal and inter-individual variation in fecal bacterial load and antimicrobial resistance genes (ARGs) burden in 15 pigs across 8 sampling weeks. **(A)** Bar plots of *16S rRNA* gene copies depicting bacterial biomass per pig. **(B)** Stacked bar plots of total ARG copies per pig, with colors representing individual ARGs contributing to the cumulative burden. Log-transformed copy numbers are shown for comparison across individuals and weeks. Substantial heterogeneity is observed both between pigs within the same week and longitudinally within pigs across weeks, indicating dynamic resistome fluctuations despite shared housing and management. Missing samples in Weeks 1, 4, and 6 were due to unsuccessful fecal collection, and some pigs were relocated to the finisher barn by Week 8.

**Figure 4:**
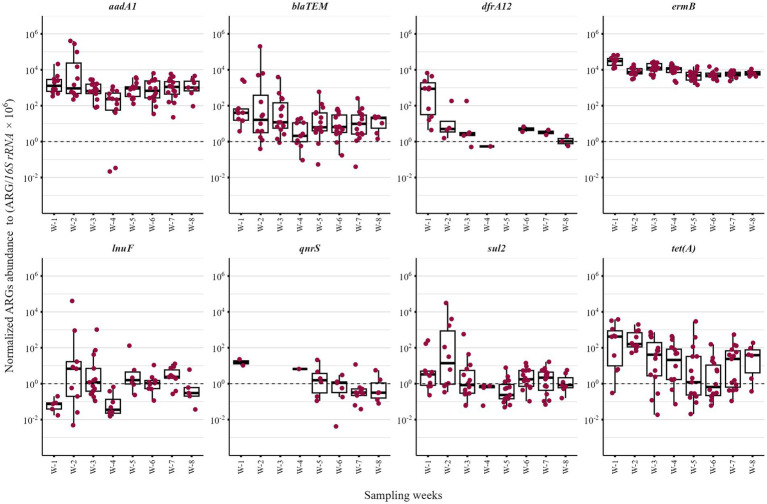
Temporal dynamics and inter-individual variability of antimicrobial resistance gene (ARG) normalized abundance (ARG/*16S rRNA* × 10^6^) across eight sampling weeks in weaner pigs. Each point represents the normalized abundance from an individual pig, and boxplots show the distribution within each week. The *y*-axis is log₁₀-scaled for comparability across genes, and faceted panels depict individual ARGs using a consistent scale. Trends over time illustrate large fluctuations in several genes, reflecting unstable ARG carriage at the individual level. The dashed horizontal line (*y* = 1) marks the threshold of normalized abundance.

Substantial intra-cohort heterogeneity in fecal resistome (gene copies) was observed among pigs under identical housing conditions, while relatively stable *16S rRNA* gene levels confirmed that this variability reflected ARG dynamics rather than total bacterial load (Figure 3). In contrast, pen-floor samples showed lower variability (Supplementary Figure S2), suggesting a more consistent ARG presence at the pen level. Both the number and normalized abundances of ARGs varied widely among and within individual weaner pigs over successive weeks, with particularly large variation in week two that narrowed by week four (Figure 4), indicating dynamic shifts in ARG carriage over time. Mixed-effects modeling further supported these observations, with low intraclass correlation coefficients (ICC < 0.06) indicating that the majority of variance in fecal ARG abundances occurred within individuals over time, highlighting substantial intra-cohort heterogeneity. Additionally, normalized gene abundances in weaner pigs did not differ by maternal origin (Kruskal–Wallis *χ*^2^ = 7.18, df = 4, *p* = 0.13). Pairwise Wilcoxon comparisons were non-significant after multiple-testing correction (all adjusted *p* > 0.05) and showed small effect sizes (rank-biserial 0.011–0.17), indicating no significant difference across litters.

### 3.4 ARG abundance was unaffected by treatment but varied with age

**Figure 5:**
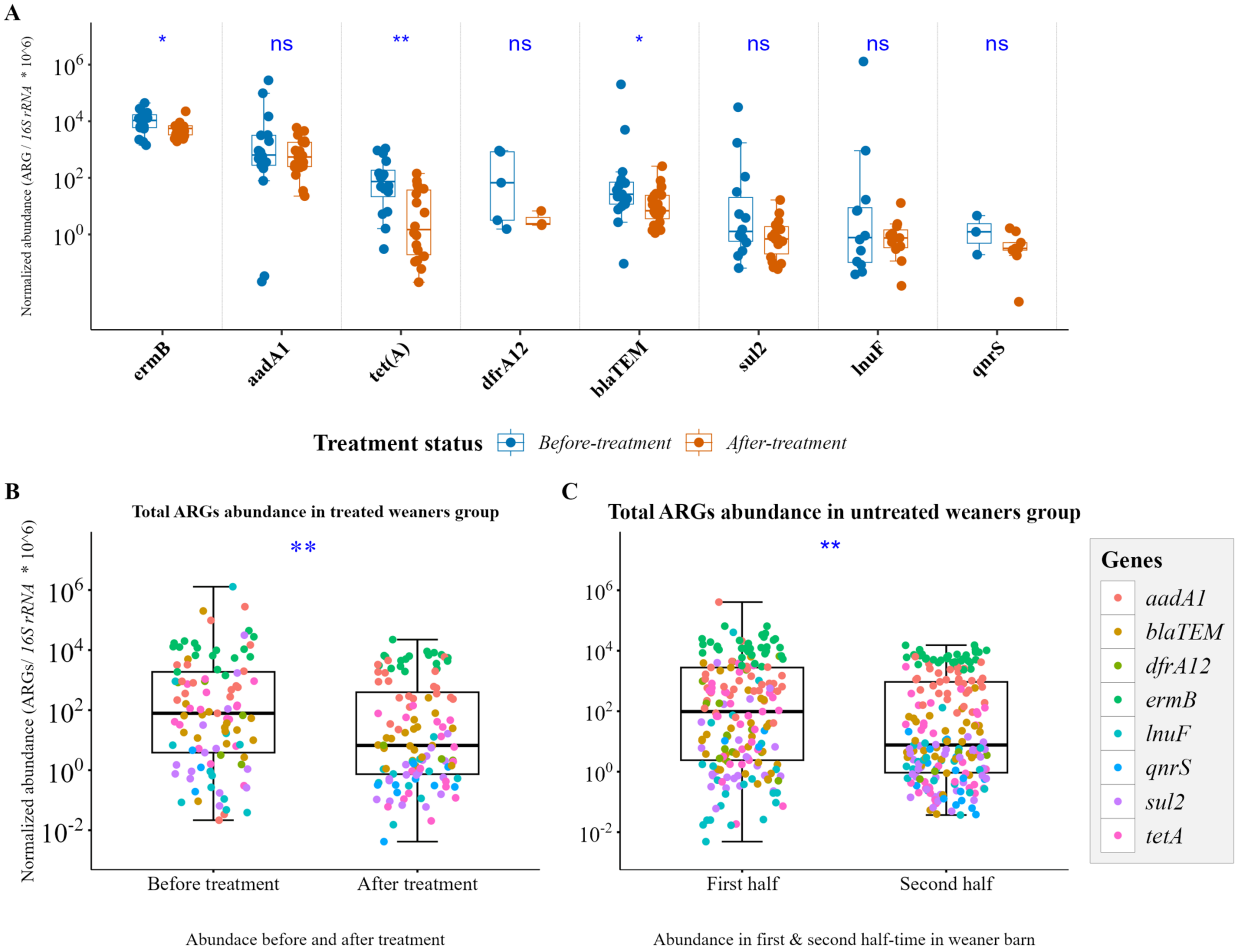
Changes in antimicrobial resistance genes (ARGs) in weaner pigs with and without antibiotic exposure. Each boxplot represents the distribution of normalized ARG abundance across pigs. **(A)** Individual ARG abundances showed slight reduction in a few specific resistance genes after antibiotic treatment, suggesting a transient effect rather than a sustained increase. **(B)** Total ARG burden in treated pigs before and after treatment. **(C)** Total ARG burden in untreated pigs during early (Weeks 1–4) vs. late (Weeks 5–8) stages, indicating that age or other temporal factors, rather than antibiotic treatment, largely drove these changes. Significance was assessed using the Wilcoxon rank-sum test for all pairwise comparisons.

Five of the 15 monitored weaners received amoxicillin for tail-bite injuries (15 mg/kg IM for 3 days): two in Week 3 and three in Week 6 (Supplementary Table S6). A significant reductions in *bla*_TEM_, *erm*(B), and *tet*(A) were observed post-treatment (Figure 5A). However, a similar decline in total ARGs was also seen in treated pigs before and after treatment (Figure 5B), as well as in untreated pigs between the first and second halves of the study period (Figure 5C), suggesting that the observed changes may be influenced by time- or age-related effects rather than treatment alone. ARG levels did not differ by sow of origin overall; however, weaners from a lincomycin-treated sow showed relatively higher ARG abundance early in the study, with *lnu*(F) sharply increasing after week 1 (Supplementary Figure S3). In general, antibiotic exposure (therapeutic) in this study had minimal impact, aside from slightly elevated *bla*_TEM_ in exposed pigs (Supplementary Figure S4). Additionally, ARG abundance was highest during the first week when pigs transitioned to Primastart feed and supplements (Supplementary Table S6; Supplementary Figure S5), indicating diet change may have significant influence on resistome profiles.

### 3.5 Higher ARG abundance in pen-floor than in individual samples

**Figure 6:**
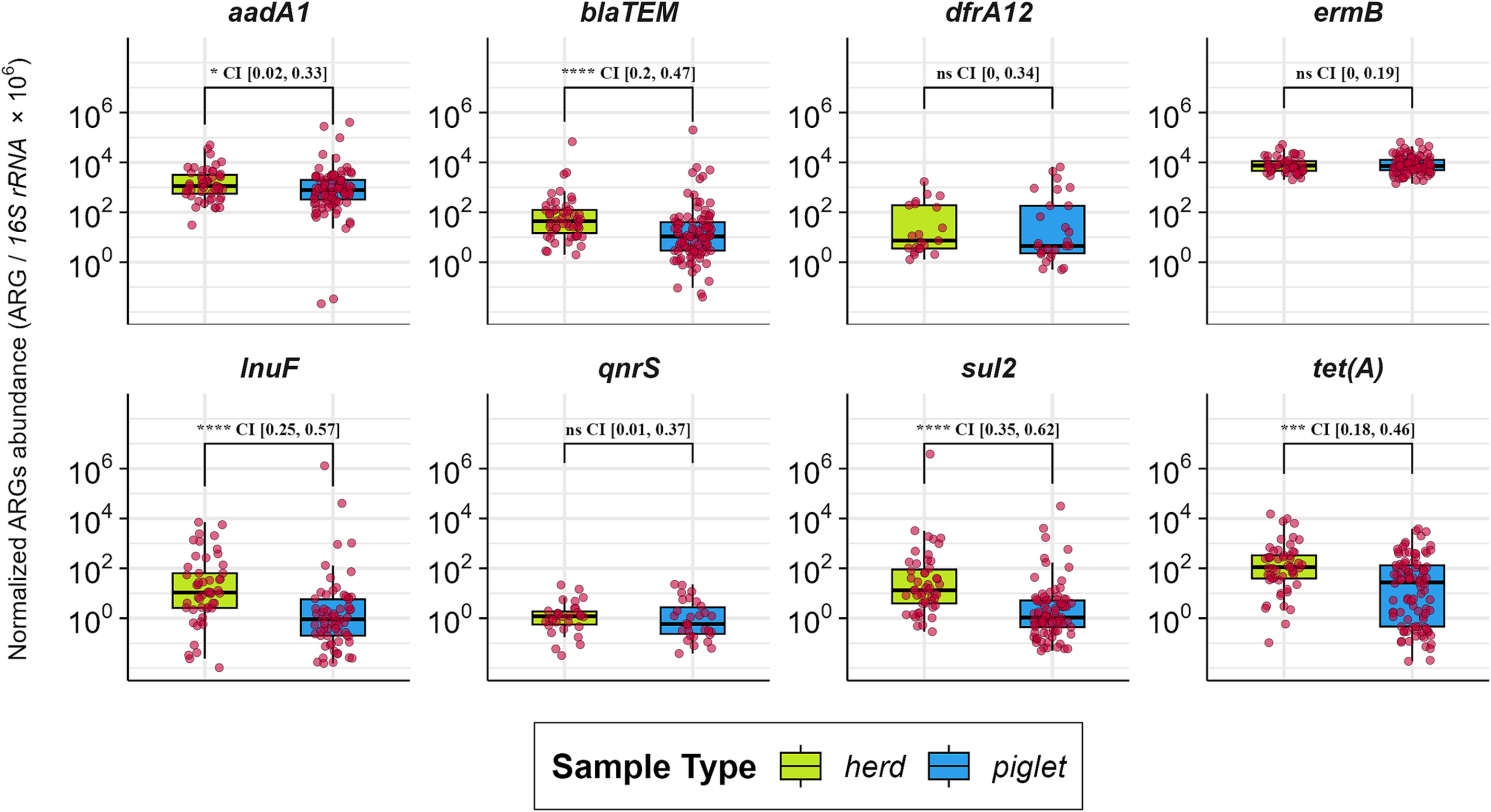
Comparison of normalized antimicrobial resistance gene (ARG) abundances between individual weaner fecal samples (blue) and pooled pen-floor fecal samples (green). Red points represent abundance values from each sample. Pooled pen-floor samples showed significantly higher ARG abundance than individual pig samples (*p* < 0.05), indicating that resistome profiles at the herd level cannot be reliably inferred from individual animals alone.

**Figure 7:**
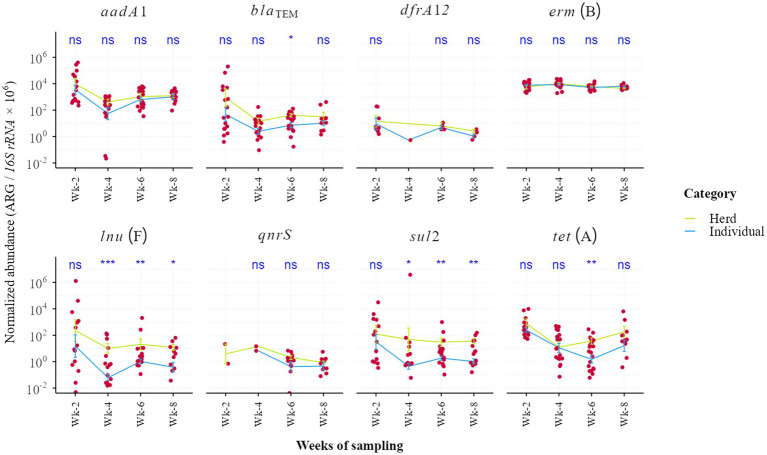
Temporal changes in antimicrobial resistance gene (ARG) abundance across 8 weeks of weaner pigs in flat deck: pen-pooled herd (orange) and individual fecal samples (blue). Mean values with standard error are shown. While *erm*(B) exhibited stable and comparable trends between sampling scales, other ARGs fluctuated more strongly over time. From week 4th onward, herd-level abundances became significantly higher in *lnu*(F) and *sul2* than those in individuals (Wilcoxon rank-sum test, *p* < 0.05), indicating amplification of resistome signals in the pen environment following fecal deposition.

Comparison of 53 pooled pen-floor fecal samples with 103 individual weaner samples revealed significantly higher abundances of key ARGs—including *bla*_TEM_, *lnu*(F), *sul2*, *tet*(A), and *aadA1*—in the pooled samples (*p* < 0.05; Figure 6). These results demonstrate that herd-level resistome profiles cannot be reliably inferred from individual animals alone. Moreover, temporal trends in *erm*(B) abundance were generally consistent between individual pigs and pooled herd samples across the sampling weeks (Figure 7). Conversely, other ARGs exhibited greater fluctuation over time. Significant differences between herd-level and individual ARGs abundance in many weeks emerged from Week 3 onward (*p* < 0.05), with *aadA1*, *bla*_TEM_, *lnu*(F), *sul2*, and *tet*(A) consistently higher in pooled samples (Figure 7).

### 3.6 Longitudinal trends of ARGs in individual fecal samples

**Figure 8:**
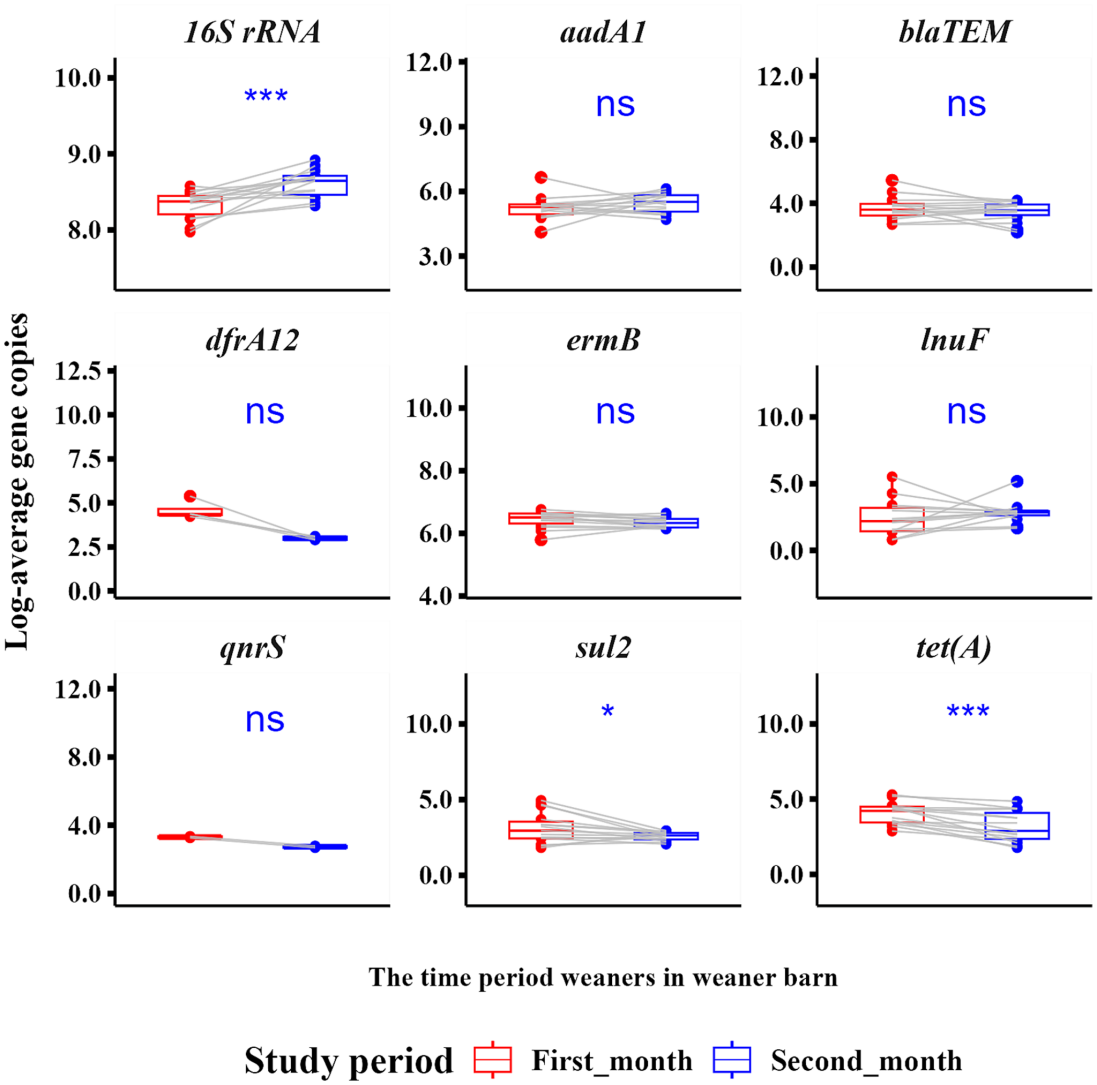
Pairwise boxplots displaying temporal changes in antimicrobial resistance genes (ARGs)and 16S rRNA gene copies in individual weaner pigs averaged over the early weaning phase (Weeks 1–4) and the post-adaptation phase (Weeks 5–8) of their stay in the weaner barn. Lines connect paired data points for each pig, with red representing the early phase and blue the later phase. This visualization highlights temporal changes in ARGs gene copies as pigs transition from dietary and environmental adjustment to increased microbiome stability. Statistically significant differences between phases were assessed using a paired *t*-test.

For temporal analysis of individual fecal samples, the monitoring period was segmented into an early weaning phase (Weeks 1–4) and a later post-adaptation phase (Weeks 5–8), reflecting the transition from dietary and environmental adjustment to greater microbiome stability (Figure 8). The *16S rRNA* gene copies increased significantly over time, indicating microbial growth, whereas *sul2* and *tet*(A) declined in the second half of the study (Figure 8).

## 4 Discussion

Systematic surveillance of AMR in livestock is essential—not only for monitoring trends and identifying emerging threats but also for guiding proper use of antimicrobials and supporting evidence-based policy decisions (34, 35). In this study, ARGs in weaner pigs were assessed using real-time PCR over an eight-week monitoring period in weaner barns. High-throughput SmartChip analysis of pooled weaner pig feces identified ARGs spanning all major antibiotic classes, predominantly *β*-lactam (*n* = 39), aminoglycoside (*n* = 34), multidrug resistance (MDR; *n* = 28), and MLSB (*n* = 22) genes. This underscores pigs as major reservoirs of ARGs capable of persisting in the gut microbiota and disseminating into the environment (36–38). Tyrrell et al. (39) reported comparable findings in weaner pig manure, while Atanasova et al. (40) identified relatively fewer *β*-lactam-resistance genes (*n* = 5) but similar levels of aminoglycoside (*n* = 28), MDR (*n* = 29), and MLSB (*n* = 25) genes in poultry manure, indicating host- and management-related differences in ARG distribution. Among bacterial taxonomic markers, *E. coli* was the most abundant, suggesting its potential role in the maintenance and dissemination of ARGs (41). In contrast, Staphylococcus markers were uncommon, indicating likely external contamination from the animals’ skin (42).

The SmartChip system enables the detection of 100 of ARGs in complex environmental matrices with high sensitivity, simultaneously identifying a broad range of genes, including those present at low abundance (22, 43). However, we found some discrepancies between the standard qPCR and smartChip in the detection of certain genes (Supplementary Table S2), which might be associated with reagent chemistries or detection conditions. Anyhow, some genes were detected by smartChip, but not by qPCR, despite using the same primer sequence, and this might be associated with differences in the technologies of the platform and differences in cycling conditions, such as initial denaturation time and selection of variable CT cut-off values (22, 23).

Analysis of individual pig feces by qPCR method demonstrated pronounced variability in antimicrobial resistance gene (ARG) abundance across animals, despite standardized environmental and husbandry conditions, while 16S rRNA gene copy numbers remained relatively stable. Notable differences were seen in *dfrA12*, *lnu*(F), *bla*_TEM_, *tet*(A), and *sul2*. Host-specific factors, including immune status, gut physiology, and mucosal integrity, also had impact on the colonization dynamics of resistant strains and ARG acquisition (37, 44, 45). Additionally, stochastic events such as colonization by specific strains, horizontal gene transfer, and microbiota shifts during weaning also mitigate ARG variability (46, 47). Maternal transmission may also effect early ARG carriage; however, sow identity did not significantly predict ARG abundance in this study, suggesting that individual colonization and stochastic factors are dominant determinants (48).

The relative abundances of *erm*(B), *aadA1*, and *tet*(A) were found to be very high in comparison to the other ARGs. By contrast, *qnrS* is only found in a small number of pigs, emphasizing how ineffectively this gene spreads between pigs. Wang et al. (49) and Petrin et al. (50) indicated a wide dissemination of *erm*(B) and *tet*(A), showing an effective maintenance among bacteria and environmental persistence. Simultaneously, other ARGs consistently exhibit high variability, indicating a smaller host range or hosts that are more susceptible to environmental or physiological influences. Despite not being exposed to antimicrobials, the genes that conferred resistance to tetracycline and macrolide stayed largely stable until 140 days, when pigs reached market weight (37). Some ARGs persist at high levels regardless of antibiotic treatment or microbiome changes, suggesting widespread dissemination throughout microbial populations (27).

The impact of therapeutic antibiotic use on ARG dynamics in weaner pigs appears complex and gene-specific. In spite of direct exposure to penicillin and indirect exposure to lincomycin in a subset of pigs, only minor, non-significant increases in *bla*_CTX-M_ and *lnu*(F) were detected. This observation is consistent with previous reports indicating that short-term antimicrobial treatments induce minimal or transient resistome changes (14, 51). Similarly, Van Gompel et al. (52) reported no strong association between *β*-lactam use and ARG abundance in young pigs. One explanation may be that penicillin exerts weaker selection pressure for ESBL-producing *Enterobacteriaceae* compared with higher-tier *β*-lactams, as reflected by persistent *bla*_CTX-M_ enrichment following cephalosporin use but only transient effects with amoxicillin (53). More broadly, ARG selection is influenced by multiple factors, including antibiotic class, exposure intensity, administration strategy, and microbiome ecology (24, 26, 28), and the findings suggest that appropriately managed therapeutic amoxicillin use exerts limited impact on resistome dynamics in weaner pig.

While these findings are consistent with EU regulations (Regulation (EU) 2019/6) that emphasize targeted antimicrobial stewardship (54), medical records revealed that five of the 15 pigs monitored longitudinally required therapeutic amoxicillin, primarily to treat tail and ear lesions. This outcome underscores the importance of strengthening management strategies to prevent such injurious behaviors and thereby reduce reliance on antimicrobials. It also reflects EU policy, which prohibits routine antibiotic use and stresses that antimicrobials should not serve as a substitute for poor hygiene or farm management.

The pooled floor (herd) samples exhibited significantly higher loads of AGRs compared with the fresh fecal samples from individual pigs. Resistance may persist from bacteria already present in the pen (55) or originate from unassigned pigs. Unlike individual fecal samples collected immediately after defecation, pooled samples were derived from pre-existing feces of unknown age and excretion time, potentially altering microbial composition (38, 56). Furthermore, accumulated pen floor feces may provide a favorable environment for microbial interactions, facilitating more efficient exchange of resistant genes through horizontal gene transfer (HGT) (24, 57). In addition, residual antibiotics or cleaning agents retained on floors can create selective pressure that promotes the persistence of ARG-carrying bacteria (58). Furthermore, the elevated abundance of ARGs in pooled samples demonstrates that pooled sampling reliably captures the resistome while offering a practical and cost-efficient startergy to resistance surveillance, achieved through streamlined sampling and reduced analytical effort (18, 20, 59).

The modest increase in *16S rRNA* gene levels in weaner pig feces over time indicates progressive maturation and stabilization of the gut microbiome. This observation is consistent with Gaire et al. (29), who reported that the fecal microbiome in female pigs exhibited reproducible individual changes and greater diversity with age. Conversely, the gradual decline in ARG abundance, which plateaued for certain genes toward the later weeks, likely reflects the establishment of microbial communities, a trend well-documented by Gaire et al. (60), and potentially linked to age. The reduction in *tet*(A) and *sul2* genes observed in this study aligns with previous reports of decreased tetracycline- and sulfonamide-resistance genes in older pigs or followed reduced antimicrobial exposure (14, 61).

High-throughput SmartChip screening revealed a broad spectrum of clinically significant ARGs, including carbapenemases (*bla*_NDM_, *bla*_VIM_, *bla*_GES_), *bla*_CTX-M-8_, *bla*_CMY-2_, and *mec*A. Environmental vectors such as flies and dust contribute to the ARG transmission both within and beyond the farm environment (62, 63). Their occurrence across animal and environmental samples underscores the One Health dimension perspective of AMR, highlighting the potential of ARG transfer to humans through occupational exposure, the food chain, and environmental contamination (64, 65).

In spite of the valuable insights provided, several additional factors should be considered when interpreting these findings. This study was carried out in a single commercial farm with a relatively small cohort of pigs, which may limit the generalizability of the findings. Methodological limitations added further uncertainty: several ARGs initially detected using SmartChip could not be validated by qPCR, and the unusually low ARG levels observed during Week 2 in two pigs most likely represent analytical variability rather than genuine biological differences. Moreover, the absence of sequencing data limited the ability to assess microbiome changes that may influence resistome dynamics. Nevertheless, this study provides an initial impression of individual ARG variability in weaner pigs and demonstrates that herd samples are at least as informative as, if not more informative than, individual samples. Future research incorporating larger, multi-farm cohorts and integrated microbiome–resistome analyses will be essential to validate and extend these results.

## 5 Conclusion

Stochastic factors, including differential exposure, microbial colonization, and environmental influences, may drive the development and acquisition of resistance genes, resulting in highly variable ARG profiles among weaner pigs and even within the same individual over time. Administration of prescribed amounts of therapeutic antibiotics did not necessarily increase ARG levels in treated pigs. Although ARG abundances fluctuate during the early weeks—likely due to dietary shifts, host-specific factors, and environmental factors—they generally decrease and stabilize over time. Hence, future studies should integrate microbiome–resistome dynamics analyses with dietary interventions to evaluate their impact on stabilizing ARG profiles and mitigating resistance development in weaner pigs.

## Data Availability

The original contributions presented in the study are included in the article/Supplementary material, further inquiries can be directed to the corresponding author.

## References

[1] Kim SW Gormley A Jang KB Duarte ME. Current status of global pig production: an overview and research trends. Anim Biosci. (2024) 37:719–29. doi: 10.5713/ab.23.0367, 37946421 PMC11016693

[2] Lekagul A Tangcharoensathien V Yeung S. Patterns of antibiotic use in global pig production: a systematic review. Vet Anim Sci. (2019) 7:100058. doi: 10.1016/j.vas.2019.100058, 32734079 PMC7386699

[3] E. F. S. A, E. C. for D. P. and C. The European Union summary report on antimicrobial resistance in zoonotic and indicator bacteria from humans, animals and food in 2021–2022. EFSA J. (2024) 22:e8583. doi: 10.2903/j.efsa.2024.8583, 38419967 PMC10900121

[4] EFSA Panel on Biological Hazards (BIOHAZ) Koutsoumanis K Allende A Álvarez-Ordóñez A Bolton D Bover-Cid S . Role played by the environment in the emergence and spread of antimicrobial resistance (AMR) through the food chain. EFSA J. (2021) 19:e06651. doi: 10.2903/j.efsa.2021.6651, 34178158 PMC8210462

[5] Graham DW Bergeron G Bourassa MW Dickson J Gomes F Howe A . Complexities in understanding antimicrobial resistance across domesticated animal, human, and environmental systems. Ann N Y Acad Sci. (2019) 1441:17–30. doi: 10.1111/nyas.14036, 30924539 PMC6850694

[6] Bonzelett C Schnepf A Hartmann M Käsbohrer A Kreienbrock L. Use of antimicrobials by class in pigs in Germany—a longitudinal description considering different international categorisation systems. Antibiotics. (2022) 11. doi: 10.3390/antibiotics11121833, 36551491 PMC9774131

[7] Dewulf J Joosten P Chantziaras I Bernaerdt E Vanderhaeghen W Postma M . Antibiotic use in European pig production: less is more. Antibiotics (Basel). (2022) 11. doi: 10.3390/antibiotics11111493, 36358148 PMC9686698

[8] Hemme M Ruddat I Hartmann M Werner N van Rennings L Käsbohrer A . Antibiotic use on German pig farms - a longitudinal analysis for 2011, 2013 and 2014. PLoS One. (2018) 13:e0199592–e0199592. doi: 10.1371/journal.pone.0199592, 29969477 PMC6029768

[9] Rennings LV Münchhausen CV Ottilie H Hartmann M. Cross-sectional study on antibiotic usage in pigs in Germany. PLoS One. (2015) 10. doi: 10.1371/journal.pone.0119114, 25785688 PMC4364977

[10] Sarrazin S Joosten P Van Gompel L Luiken REC Mevius DJ Wagenaar JA . Quantitative and qualitative analysis of antimicrobial usage patterns in 180 selected farrow-to-finish pig farms from nine European countries based on single batch and purchase data. J Antimicrob Chemother. (2019) 74:807–16. doi: 10.1093/jac/dky503, 30544242

[11] Schaekel F May T Seiler J Hartmann M Kreienbrock L. Antibiotic drug usage in pigs in Germany-are the class profiles changing? PLoS One. (2017) 12:e0182661. doi: 10.1371/journal.pone.0182661, 28841685 PMC5571922

[12] Munk P Knudsen BE Lukjancenko O Duarte ASR Van Gompel L Luiken REC . Abundance and diversity of the faecal resistome in slaughter pigs and broilers in nine European countries. Nat Microbiol. (2018) 3:898–908. doi: 10.1038/s41564-018-0192-9, 30038308

[13] Schokker D Zhang J Zhang L Vastenhouw SA Heilig HGHJ Smidt H . Early-life environmental variation affects intestinal microbiota and immune development in new-born piglets. PLoS One. (2014) 9:e100040. doi: 10.1371/journal.pone.0100040, 24941112 PMC4062469

[14] Tams KW Larsen I Hansen JE Spiegelhauer H Strøm-Hansen AD Rasmussen S . The effects of antibiotic use on the dynamics of the microbiome and resistome in pigs. Anim Microbiome. (2023) 5. doi: 10.1186/s42523-023-00258-4, 37605221 PMC10440943

[15] Nunan C. Ending routine farm antibiotic use in Europe. (2022). Available online at: https://epha.org/ending-routine-farm-antibiotic-use/ (Accessed August 21, 2025).

[16] Steen AD Crits-Christoph A Carini P DeAngelis KM Fierer N Lloyd KG . High proportions of bacteria and archaea across most biomes remain uncultured. ISME J. (2019) 13:3126–30. doi: 10.1038/s41396-019-0484-y, 31388130 PMC6863901

[17] Wylensek D Hitch TCA Riedel T Afrizal A Kumar N Wortmann E . A collection of bacterial isolates from the pig intestine reveals functional and taxonomic diversity. Nat Commun. (2020) 11:6389. doi: 10.1038/s41467-020-19929-w, 33319778 PMC7738495

[18] Clasen J Mellerup A Olsen JE Angen Ø Folkesson A Halasa T . Determining the optimal number of individual samples to pool for quantification of average herd levels of antimicrobial resistance genes in Danish pig herds using high-throughput qPCR. Vet Microbiol. (2016) 189. doi: 10.1016/j.vetmic.2016.04.017, 27259826

[19] Ferreira C Otani S Aarestrup FM Manaia CM. Quantitative PCR versus metagenomics for monitoring antibiotic resistance genes: balancing high sensitivity and broad coverage. FEMS Microbes. (2023) 4:1–7. doi: 10.1093/femsmc/xtad008, 37333442 PMC10117749

[20] Schmidt G Mellerup A Christiansen LE Ståhl M Olsen JE Angen Ø. Sampling and pooling methods for capturing herd level antibiotic resistance in swine feces using qPCR and CFU approaches. PLoS One. (2015) 10. doi: 10.1371/journal.pone.0131672, 26114765 PMC4483237

[21] Taylor W Bohm K Dyet K Weaver L Pattis I. Comparative analysis of qPCR and metagenomics for detecting antimicrobial resistance in wastewater: a case study. BMC Res Notes. (2025) 18:5. doi: 10.1186/s13104-024-07027-9, 39773654 PMC11705827

[22] Liu X Xiao P Guo Y Liu L Yang J. The impacts of different high-throughput profiling approaches on the understanding of bacterial antibiotic resistance genes in a freshwater reservoir. Sci Total Environ. (2019) 693:133585. doi: 10.1016/j.scitotenv.2019.133585, 31377359

[23] Waseem H Jameel S Ali J Ur Rehman HS Tauseef I Farooq U . Contributions and challenges of high-throughput qPCR for determining antimicrobial resistance in the environment: a critical review. Molecules. (2019) 24. doi: 10.3390/molecules24010163, 30609875 PMC6337382

[24] Ghanbari M Klose V Crispie F Cotter PD. The dynamics of the antibiotic resistome in the feces of freshly weaned pigs following therapeutic administration of oxytetracycline. Sci Rep. (2019) 9:4062. doi: 10.1038/s41598-019-40496-8, 30858509 PMC6411716

[25] Græsbøll K Larsen I Clasen J Birkegård AC Nielsen JP Christiansen LE . Effect of tetracycline treatment regimens on antibiotic resistance gene selection over time in nursery pigs. BMC Microbiol. (2019) 19:269. doi: 10.1186/s12866-019-1619-z, 31791243 PMC6889206

[26] Herrero-Fresno A Olsen JE. Effect of ampicillin, cephalexin, ceftiofur and tetracycline treatment on selection of resistant coliforms in a swine faecal microcosmos. J Appl Microbiol. (2020) 129:1238–47. doi: 10.1111/jam.14721 32430970

[27] Pollock J Muwonge A Hutchings MR Mainda G Bronsvoort BM Gally DL . Resistance to change: AMR gene dynamics on a commercial pig farm with high antimicrobial usage. Sci Rep. (2020) 10:1–10. doi: 10.1038/s41598-020-58659-3 32015392 PMC6997390

[28] Zeineldin M Megahed A Burton B Blair B Aldridge B Lowe JF. Effect of single dose of antimicrobial administration at birth on fecal microbiota development and prevalence of antimicrobial resistance genes in piglets. Front Microbiol. (2019) 10:1414. doi: 10.3389/fmicb.2019.01414, 31275295 PMC6593251

[29] Gaire TN Scott HM Noyes NR Ericsson AC Tokach MD William H . Temporal dynamics of the fecal microbiome in female pigs from early life through estrus, parturition, and weaning of the first litter of piglets. Anim Microbiome. (2024) 6:7. doi: 10.1186/s42523-024-00294-8, 38383422 PMC10882843

[30] Bassitta R. [Studies on the selection of resistance genes in Bavarian pig farms and on the transmission of antibiotic-resistant *E. coli* between animals and humans]. Dissertation, LMU München: Faculty of Veterinary Medicine (2016). doi: 10.5282/edoc.19268

[31] Jaleta M Junker V Kolte B Börger M Werner D Dolsdorf C . Improvements of weaned pigs barn hygiene to reduce the spread of antimicrobial resistance. Front Microbiol. (2024) 15. doi: 10.3389/fmicb.2024.1393923, 38812683 PMC11135127

[32] Costea PI Zeller G Sunagawa S Pelletier E Alberti A Levenez F . Towards standards for human fecal sample processing in metagenomic studies. Nat Biotechnol. (2017) 35:1069–76. doi: 10.1038/nbt.3960, 28967887

[33] Stedtfeld RD Guo X Stedtfeld TM Sheng H Williams MR Hauschild K . Primer set 2.0 for highly parallel qPCR array targeting antibiotic resistance genes and mobile genetic elements. FEMS Microbiol Ecol. (2018) 94:fiy130. doi: 10.1093/femsec/fiy130, 30052926 PMC7250373

[34] Food and Agriculture Organization of the United Nations (FAO). Surveillance and monitoring—antimicrobial resistance. (2023). Available online at: https://www.fao.org/antimicrobial-resistance/key-sectors/surveillance-and-monitoring/en/ (Accessed August 21, 2025).

[35] World Organisation for Animal Health (WOAH). editor. Terrestrial Animal Health Code. Chapter 6.8: Harmonisation of national antimicrobial resistance surveillance and monitoring programmes. Paris: WOAH. (2024). Available online at: https://www.woah.org/fileadmin/Home/eng/Health_standards/tahc/2024/en_chapitre_antibio_harmonisation.htm

[36] Checcucci A Trevisi P Luise D Modesto M Blasioli S Braschi I . Exploring the animal waste resistome: the spread of antimicrobial resistance genes through the use of livestock manure. Front Microbiol. (2020) 11. doi: 10.3389/fmicb.2020.01416, 32793126 PMC7387501

[37] Holman DB Gzyl KE Mou KT Allen HK. Weaning age and its effect on the development of the swine gut microbiome and resistome. mSystems. (2021) 6. doi: 10.1128/msystems.00682-21, 34812652 PMC8609972

[38] Luise D Le Sciellour M Buchet A Resmond R Clement C Rossignol MN . The fecal microbiota of piglets during weaning transition and its association with piglet growth across various farm environments. PLoS One. (2021) 16. doi: 10.1371/journal.pone.0250655, 33905437 PMC8078812

[39] Tyrrell C Do TT Leigh RJ Burgess CM Brennan FP Walsh F. Differential impact of swine, bovine, and poultry manure on the microbiome and resistome of agricultural grassland. Sci Total Environ. (2023) 886:163926. doi: 10.1016/j.scitotenv.2023.163926 37156383

[40] Atanasova A Amon T Roesler U Friese A Merle R Kabelitz T. Temporal dynamics of antimicrobial resistance gene abundances in chicken manure and anaerobic digestate. Front Antibiotics. (2025) 4:1612886. doi: 10.3389/frabi.2025.1612886 PMC12245806 40655033

[41] Rana C Vikas V Awasthi S Gautam D Vats A Rajput S . Antimicrobial resistance genes and associated mobile genetic elements in *Escherichia coli* from human, animal and environment. Chemosphere. (2024) 369:143808. doi: 10.1016/j.chemosphere.2024.143808, 39608649

[42] Linhares LL Yang M Sreevatsan S Munoz-Zanzi CA Torremorell M Davies PR. The effect of anatomic site and age on detection of *Staphylococcus aureus* in pigs. J Vet Diagn Invest. (2015) 27:55–60. doi: 10.1177/1040638714559598, 25525138

[43] Waseem H Saleem ur Rehman H Ali J Iqbal MJ Ali MI. Chapter 14 - global trends in ARGs measured by HT-qPCR platforms In: Hashmi MZ, editor. Antibiotics and antimicrobial resistance genes in the environment. Hoboken: Elsevier (2020). 206–22.

[44] Caballero-Flores G Pickard JM Núñez G. Microbiota-mediated colonization resistance: mechanisms and regulation. Nat Rev Microbiol. (2023) 21:347–60. doi: 10.1038/s41579-022-00833-7, 36539611 PMC10249723

[45] Zhou Y Fu H Yang H Wu J Chen Z Jiang H . Extensive metagenomic analysis of the porcine gut resistome to identify indicators reflecting antimicrobial resistance. Microbiome. (2022) 10:1–16. doi: 10.1186/s40168-022-01241-y 35246246 PMC8895625

[46] Rahman N McCullough T Orozco DF Walkowiak S Farzan A Shekarriz S . Genomic characterization of antimicrobial resistance and mobile genetic elements in swine gut bacteria isolated from a Canadian research farm. Anim Microbiome. (2025) 7:66. doi: 10.1186/s42523-025-00432-w, 40533851 PMC12175345

[47] Suriyaphol P Chiu JKH Yimpring N Tunsagool P Mhuantong W Chuanchuen R . Dynamics of the fecal microbiome and antimicrobial resistome in commercial piglets during the weaning period. Sci Rep. (2021) 11:18091. doi: 10.1038/s41598-021-97586-9, 34508122 PMC8433359

[48] Burow E Rostalski A Harlizius J Gangl A Simoneit C Grobbel M . Antibiotic resistance in *Escherichia coli* from pigs from birth to slaughter and its association with antibiotic treatment. Prev Vet Med. (2019) 165:52–62. doi: 10.1016/j.prevetmed.2019.02.008, 30851928

[49] Wang L Oda Y Grewal S Morrison M Michel FCJ Yu Z. Persistence of resistance to erythromycin and tetracycline in swine manure during simulated composting and lagoon treatments. Microb Ecol. (2012) 63:32–40. doi: 10.1007/s00248-011-9921-9 21811793

[50] Petrin S Patuzzi I Di Cesare A Tiengo A Sette G Biancotto G . Evaluation and quantification of antimicrobial residues and antimicrobial resistance genes in two Italian swine farms. Environ Pollut. (2019) 255:113183. doi: 10.1016/j.envpol.2019.113183, 31541814

[51] Balasubramanian R Kalmar L Holmes M Restif O. Effects of antibiotic treatment on antimicrobial resistance in pig microbiomes: a longitudinal study. Eur J Pub Health. (2021) 31:298. doi: 10.1093/eurpub/ckab165.298

[52] Van Gompel L Luiken REC Sarrazin S Munk P Knudsen BE Hansen RB . The antimicrobial resistome in relation to antimicrobial use and biosecurity in pig farming, a metagenome-wide association study in nine European countries. J Antimicrob Chemother. (2019) 74:865–76. doi: 10.1093/jac/dky518 30649386

[53] Cavaco L Abatih E Aarestrup F Guardabassi F. Selection and persistence of CTX-M-producing *Escherichia coli* in the intestinal flora of pigs treated with amoxicillin, ceftiofur, or cefquinome. Antimicrob Agents Chemother. (2008) 52:3612–6. doi: 10.1128/aac.00354-08, 18644956 PMC2565910

[54] European Parliament and Council of the European Union. 2019/6 of the European Parliament and of the Council of 11 December 2018 on veterinary medicinal products and repealing Directive 2001/82/EC (Text with EEA relevance). (2018). Available online at: http://data.europa.eu/eli/reg/2019/6/2022-01-28 (Accessed July 21, 2025).

[55] Smith RP May HE AbuOun M Stubberfield E Gilson D Chau KK . A longitudinal study reveals persistence of antimicrobial resistance on livestock farms is not due to antimicrobial usage alone. Front Microbiol. (2023) 14:1070340. doi: 10.3389/fmicb.2023.1070340, 36998408 PMC10043416

[56] Le Sciellour M Zemb O Hochu I Riquet J Gilbert H Giorgi M . Effect of chronic and acute heat challenges on fecal microbiota composition, production, and thermoregulation traits in growing pigs1,2. J Anim Sci. (2019) 97:3845–58. doi: 10.1093/jas/skz222, 31268142 PMC6735821

[57] Checcucci A Buscaroli E Modesto M Luise D Blasioli S Scarafile D . The swine waste resistome: spreading and transfer of antibiotic resistance genes in *Escherichia coli* strains and the associated microbial communities. Ecotoxicol Environ Saf. (2024) 283:116774. doi: 10.1016/j.ecoenv.2024.116774, 39053184

[58] Chee-Sanford JC Mackie RI Koike S Krapac IG Lin Y-F Yannarell AC . Fate and transport of antibiotic residues and antibiotic resistance genes following land application of manure waste. J Environ Qual. (2009) 38:1086–108. doi: 10.2134/jeq2008.0128 19398507

[59] Wagner BA Dargatz DA Salman MD Morley PS Wittum TE Keefe TJ. Comparison of sampling techniques for measuring the antimicrobial susceptibility of enteric *Escherichia coli* recovered from feedlot cattle. Am J Vet Res. (2002) 63:1662–70. doi: 10.2460/ajvr.2002.63.1662, 12492280

[60] Gaire TN Scott HM Noyes NR Ericsson AC Tokach MD Menegat MB . Age influences the temporal dynamics of microbiome and antimicrobial resistance genes among fecal bacteria in a cohort of production pigs. Anim Microbiome. (2023) 5:2. doi: 10.1186/s42523-022-00222-8, 36624546 PMC9830919

[61] Wegl G Rolinec M Nagl V Gierus M Klose V. Effect of oxytetracycline as well as an acid-based feed supplement on the prevalence and abundance of selected antibiotic resistance genes in weaned piglets. Anim Husb Dairy Vet Sci. (2017) 1. doi: 10.15761/AHDVS.1000117

[62] Behrens W Kolte B Junker V Frentrup M Dolsdorf C Börger M . Bacterial genome sequencing tracks the housefly-associated dispersal of fluoroquinolone‐ and cephalosporin‐resistant Escherichia coli from a pig farm. Environ Microbiol. (2023) 25:1174–85. doi: 10.1111/1462-2920.16352 36772962

[63] Song L Wang C Jiang G Ma J Li Y Chen H . Bioaerosol is an important transmission route of antibiotic resistance genes in pig farms. Environ Int. (2021) 154:106559. doi: 10.1016/j.envint.2021.106559 33864959

[64] Bergšpica I Kaprou G Alexa EA Prieto M Alvarez-Ordóñez A. Extended Spectrum β-Lactamase (ESBL) producing Escherichia coli in Pigs and Pork meat in the European Union. Antibiotics. (2020) 9:678. doi: 10.3390/antibiotics9100678 33036406 PMC7600538

[65] Wang Y-C He L-Y Wu H-Y Qiao L-K Huang Z Bai H . High-risk plasmid-borne resistance genes from swine farm environments infiltrate deep soil and interact with the human gut microbiome via horizontal transfer. J Hazard Mater. (2025) 496:139281. doi: 10.1016/j.jhazmat.2025.139281 40706155

