# Supplementary material for: Antimicrobial resistance genes in weaned pigs: quantitative abundance and group dynamics assessed by qPCR
Source: Front Vet Sci. 2026 Jan 5;12:1709227. doi: 10.3389/fvets.2025.1709227 (PMC12812694; doi:10.3389/fvets.2025.1709227)
Supplement: Supplementary file 1 [file Data_Sheet_1.DOCX]

Supplementary material

**Antimicrobial resistance genes in weaned pigs: Individual quantitative abundance and group dynamics, as determined by qPCR**

Megarsa Jaleta^1*^, Vera Junker^2^, Christina Hölzel^3^, Jürgen Zentek^4^, Thomas Amon^1, 5^, Ulrich Nübel^2, 6, 7^ & Tina Kabelitz^1^

^1 Leibniz Institute for Agricultural Engineering and Bioeconomy (ATB), Potsdam, Germany^

^2 Leibniz-Institute DSMZ – German Collection of Microorganisms and Cell Cultures, Braunschweig, Germany^

^3 Faculty of Agricultural and Nutritional Sciences Christian-Albrechts-University of Kiel, Kiel, Germany^

^4 Institute of Animal Nutrition, Freie Universität Berlin, Berlin, Germany^

^5 Institute for Animal Hygiene and Environmental Health (ITU), Free University Berlin, Berlin, Germany^

^6 German Center for Infection Research (DZIF), Partner Site Braunschweig-Hannover, Braunschweig, Germany^

^7 Technical University Braunschweig, Institute of Microbiology, Braunschweig, Germany^

*Corresponding author email: [mjaleta@atb-potsdam.de](mailto:mjaleta@atb-potsdam.de)

**Information about farm management**

1. **Feeding**

After arrival, weaned pigs were fed a porridge made of linseed, oat flakes, and humocarb (charcoal; 33 g per bucket) for two days. From the first day until the end of the fourth week, weaner pigs were offered a rearing feed (Feed I), Primastart (Belziger Kraftfutter GmbH, Bad Belzig, Germany), provided in flour form. Additionally, all piglets received haycobs for approximately two weeks and an electrolyte solution for two days, starting on the third day post-arrival. After four weeks, Primastart was gradually combined with another rearing feed (Feed II), Vital II (Belziger Kraftfutter GmbH, Bad Belzig, Germany), prepared in pellet form. The mixture was provided until the sixth week, after which the diet was fully transitioned to Vital II until pigs were transferred to the finisher barn. Details of the feeding strategy and the composition of both basal and supplemental feeds are presented in the supplementary material (Table S6).

1. **Vaccination and medication**

Approximately two to three weeks after the piglets arrived at the barn, they were vaccinated against Porcine Reproductive and Respiratory Syndrome virus (PRRSV) with a single 2 ml dose of Ingelvac® PRRS MLV vaccine (Boehringer Ingelheim Vetmedica GmbH, Rhein, Germany), and against Post-Weaning Multisystemic Wasting Syndrome (PMWS) with a single 1 ml dose of Ingelvac CircoFLEX® vaccine (Boehringer Ingelheim Vetmedica GmbH, Rhein, Germany). The farm veterinarian prescribes therapeutic antibiotics for pigs exhibiting clinical signs of illness. During the study period, a few treated pigs were housed in the fourth and fifth isolation pens, where they remained throughout the post-treatment phase. The research team had no involvement in the decision-making, prescription, or administration of antibiotics or other therapeutic interventions.

1. **Information related to sample collection**

Fecal samples were obtained exclusively from freshly defecated and on the pen floor of weaner pigs, ensuring no harm or distress to the animals. Fecal samples were carefully collected in sterile 120 ml propylene containers. For individual fecal sampling, marked weaners were closely observed, and as soon as one began to defecate, a collection cup was placed beneath the anus to catch the feces directly, ensuring no contact with the floor. However, if the feces fell on the pen floor before the container could be placed beneath it, the top portion of the feces was carefully removed (without touching the ground) with a sterile spatula and put in the sterile container. Note that some pigs did not have samples taken during the first, fourth, and sixth weeks because the collector was unable to watch them while they defecated, or they did not defecate during the monitoring time. Furthermore, by the eighth week, some piglets had already moved to the finisher barn, as shown in Table S4.


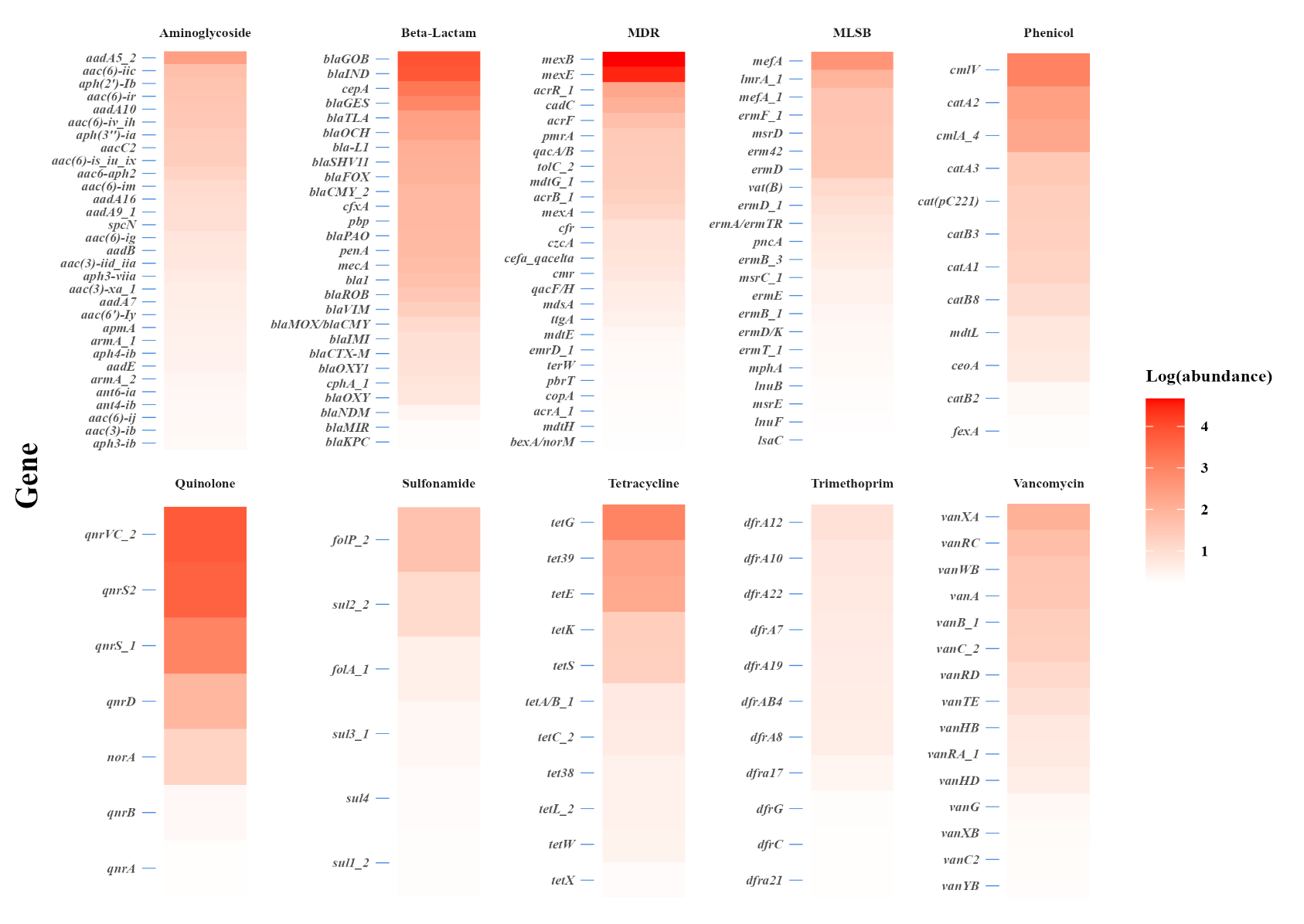


**Fig. S1**. Heatmap shows the log-transformed abundances of antimicrobial resistance genes (ARGs) detected by smartChip in two pooled pig fecal samples. Each facet indicates the resistance gene clusters into antimicrobial classes. The y-axis indicates genes categorized into functional groups. Deep red tiles show higher gene abundance, while white tile indicates lower abundance. *MDR* = multidrug resistance, *MLSB* = macrolide, lincosamide, and streptogramin.


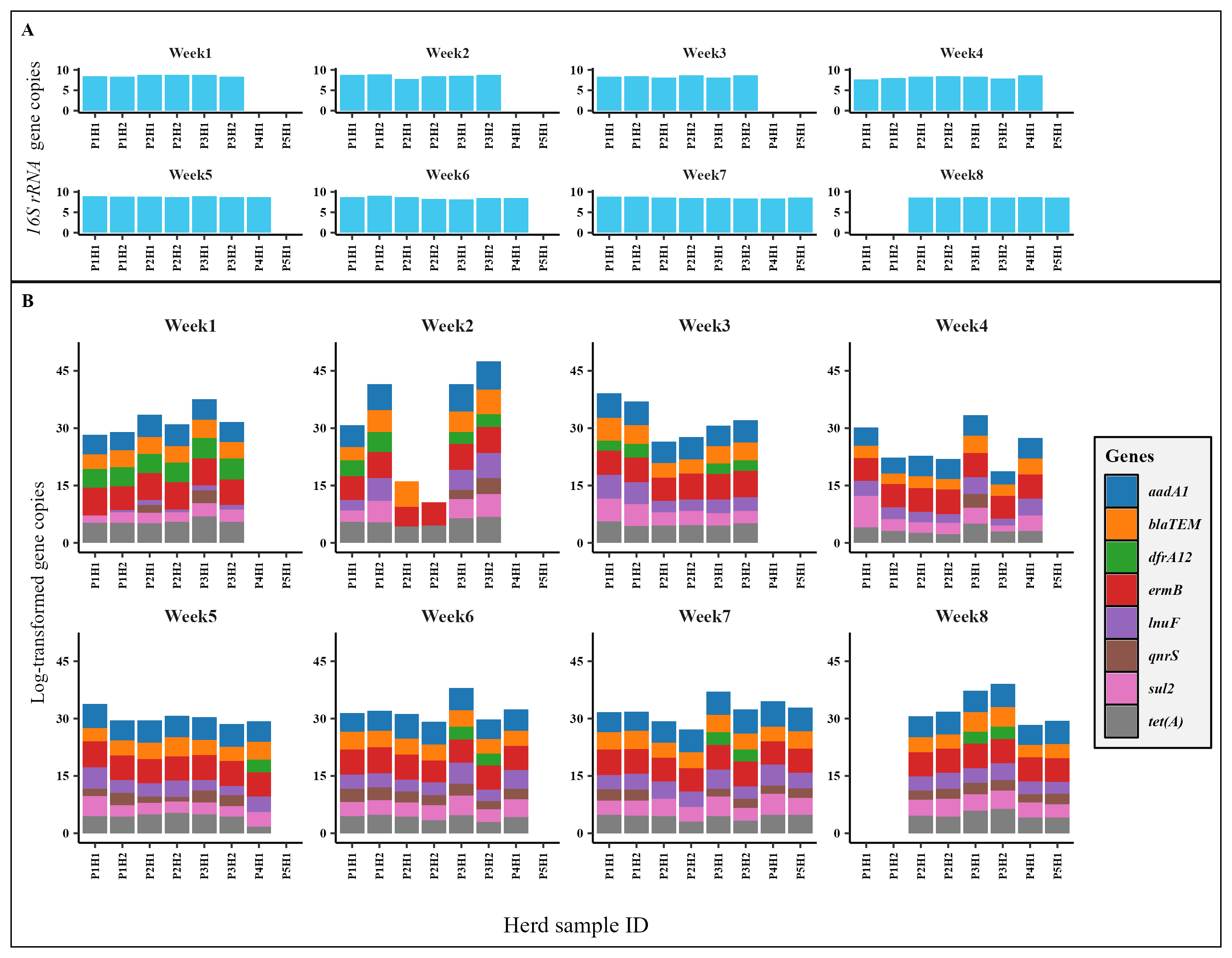


**Fig. S2**. The barplot of *16S rRNA* gene copy (**Panel A**) and stacked bar plot of log-transformed ARG copies (**Panel B**) in herd (pooled pen floor) samples in different sampling weeks. Each facet represents a specific sampling week. The y-axis represents the log-transformed abundance of gene copies, while the x-axis represents the herd sample codes (represented as P1H1 to P5H1). In **Panel B,** Different genes are color-coded, with the ARGs stacked within each pooled sample; the total bar height represents the cumulative gene copies, and the colored segments indicate the contribution of different ARGs. The use of log-transformed values ensures clearer comparisons of ARG abundance levels in the herd sample. The legend provides definitions for gene colors. ***Note***: The absence of samples P4H1 and/or P5H1 during weeks 1 to 6 suggests that the treated piglets were not isolated in the room, and the room remained empty during that period. Additionally, the samples P1H1 and P1H2 from week 8 were collected because the pigs had been moved to the finisher barns, while the other pigs in the remaining pens stayed in the weaner barns for a few extra days.


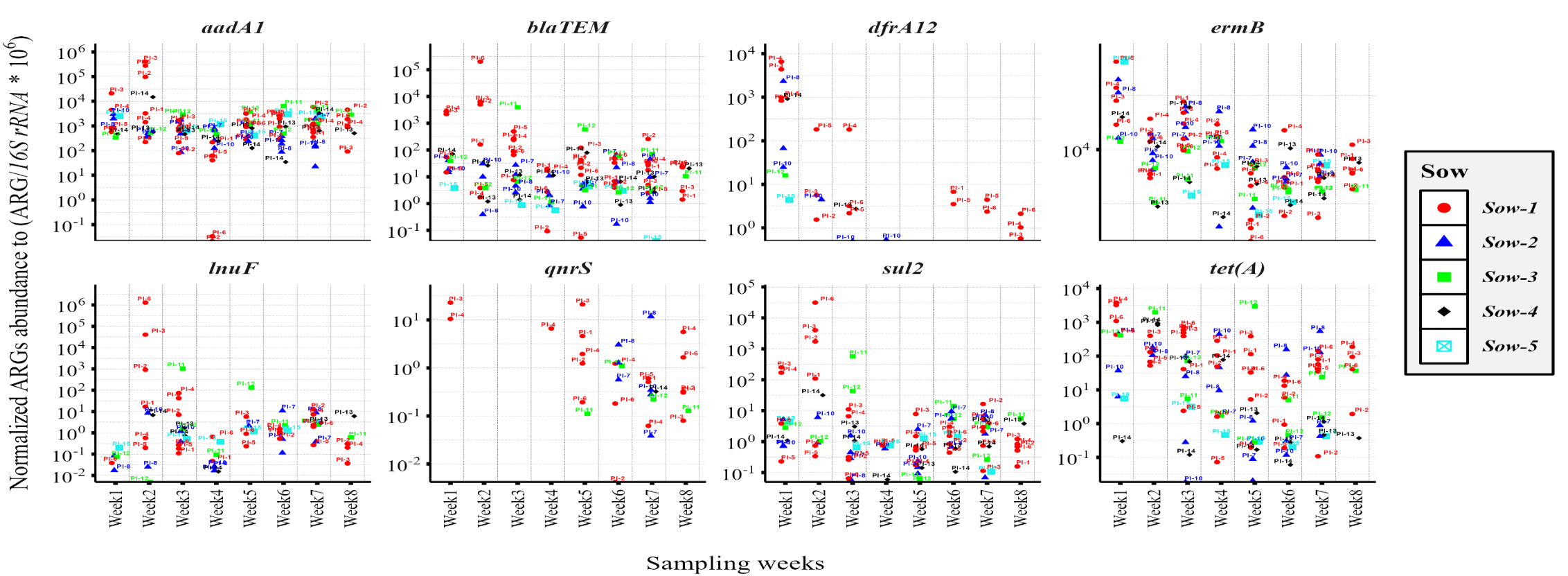


**Fig. S3**. Normalized abundance of antimicrobial resistance genes (ARGs) in weaner pigs, grouped by maternal origin. Each point represents an individual weaner pig, colored and shaped by the mother sow. The y-axis displays the normalized abundance of ARGs to *16S rRNA*, shown on a log scale. Facets represent different ARGs with free y-axis scaling. Individual weaner IDs are annotated next to each point. Vertical lines denote weekly separations, and dashed horizontal grid lines enhance readability. Note: The medical history record indicates that the Sow-1 was treated with lincomycin during the farrowing period in the breeder farm.


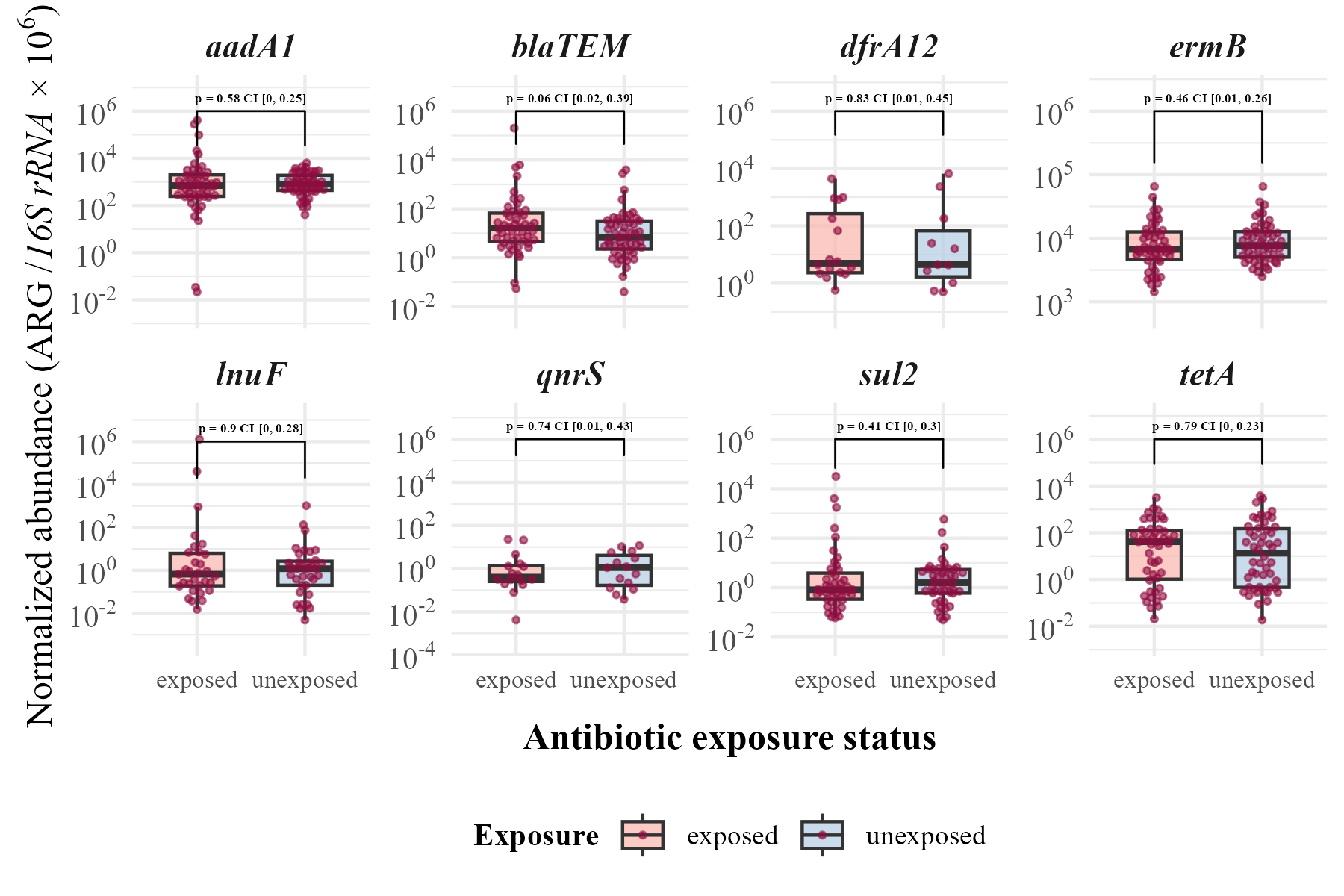


**Fig. S4**. **Comparison of normalized ARG abundance in antibiotic-exposed and unexposed weaner pigs.** Boxplots show the log-transformed normalized abundance (ARGs per 16S rRNA × 10⁶) for each antimicrobial resistance gene, comparing pigs with and without prior antibiotic exposure. Individual sample points are overlaid with jitter to display variability within groups. Statistical comparisons between exposed and unexposed groups were conducted using the *Wilcoxon test*; p-values are displayed in each panel. Each facet represents a distinct ARG, and free y-axis scaling was applied to accommodate differences in abundance levels between genes. Note: In this study, antibiotic exposure was defined as the direct administration of antibiotics to individual pigs or as the result of the mother sows' treatment during the farrowing period, then her piglets were considered exposed.


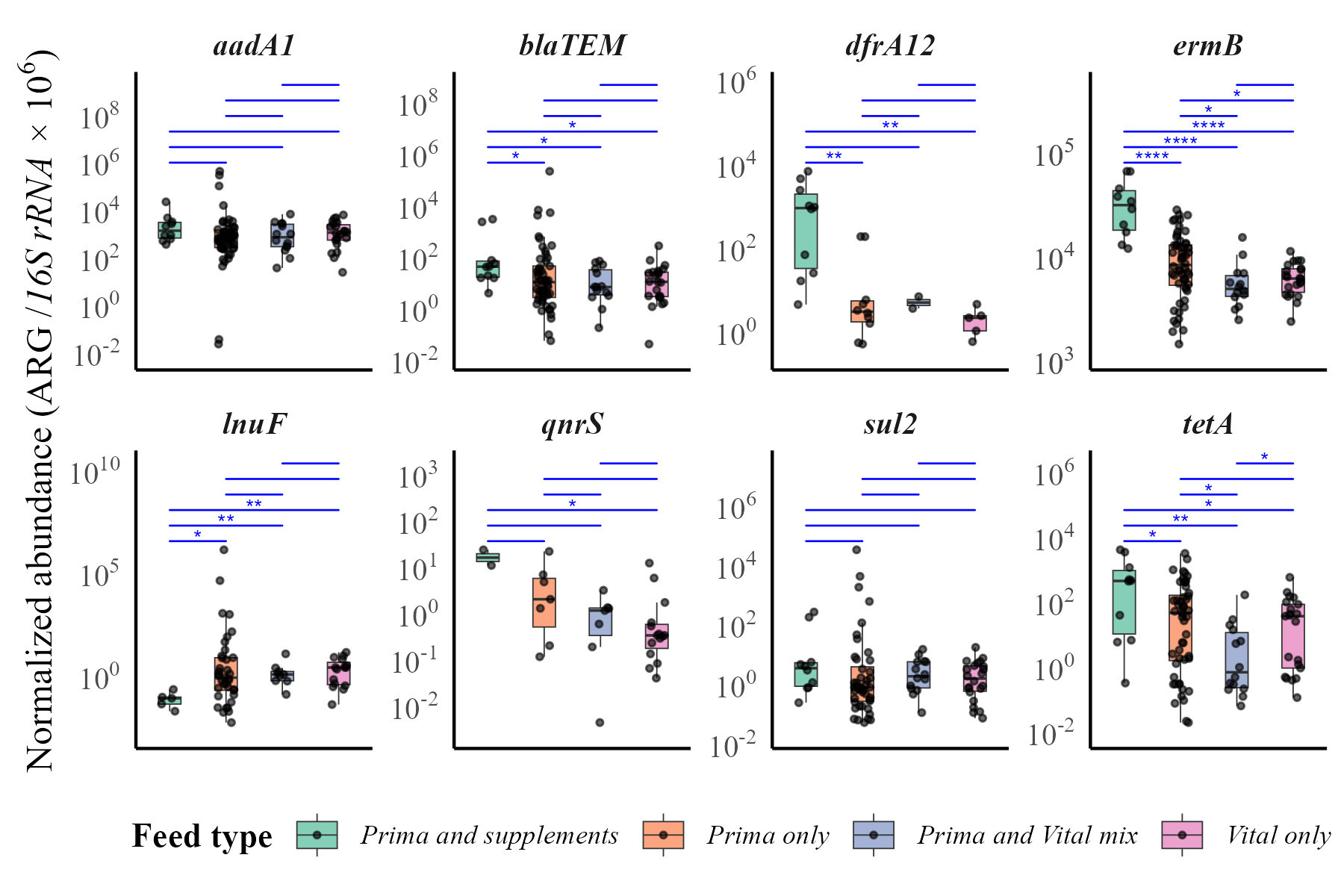


**Fig. S5**. Boxplots show the differences in normalized ARG abundance across four feed groups that weaner pigs were fed during their time in weaner barns. The Y-axis represents the distribution of weaners normalized abundance (ARGs per *16S rRNA* × 10^6^) across four feed groups. Individual data points are overlaid with jitter for visibility. Comparisons between groups were evaluated using the *Wilcoxon test*, with significance indicated by p-value labels. Each facet represents a different ARGs. The legend identifies the feed groups a description of each feed group found the Table S1_Supplemental tables.
